# Subcutaneous Infection with *Dirofilaria* spp. Nematode in Human, France

**DOI:** 10.3201/eid1911.131176

**Published:** 2013-11

**Authors:** Charles Mary, Maud Foissac, Matthieu Million, Philippe Parola, Renaud Piarroux

**Affiliations:** Assistance Publique–Hôpitaux de Marseille, Marseille, France (C. Mary, M. Foissac, M. Million, P. Parola, R. Piarroux);; Aix-Marseille Université, Marseille (C. Mary, M. Foissac, M. Million, P. Parola)

**Keywords:** subcutaneous dirofilariasis, Dirofilaria immitis, Dirofilaria repens, Dirofilaria spp., filariasis, nematodes, France, zoonoses, parasites, dirofilariasis, molecular typing, morphology

**In Response:** We agree with Eberhard (*1*) that it is difficult to make a species identification when data derived from morphologic examinations do not correlate with those of molecular diagnostics. Errors may be the result of poor indexing of sequences deposited in sequence databases or inaccurate estimation of the degree of genomic polymorphisms within a species and between closely related species. On the other hand, a morphologic difference between 2 organisms, if it is associated with only 1 characteristic, should not be considered sufficient to classify them as 2 distinct species. Such a phenotypic variation may be the result of a single mutation or deletion. Consequently, the absence of a certain character does not exclude the categorization of an organism as a given species.

Molecular identification of the *Dirofilaria* spp. worm in our clinical case was made on the basis of 2 distinct sequences, each of which exhibited marked differences between *D. immitis* and *D. repens* (*2*). The first sequence targeted internal transcribed spacer regions of ribosomal genes and revealed up to 100% homology with *D. immitis* sequences from GenBank, whereas a maximum homology of 80% was observed with *D. repens* sequences from GenBank. The second sequence targeted the cytochrome oxidase 1 gene and showed 100% homology with *D. immitis*, whereas <90% homology was observed for *D. repens*. For both analyzed targets, GenBank contained several sequences for *D. immitis* and *D. repens* that were deposited by various investigators, and all sequences yielded consistent results. Therefore, there is no basis to suggest that the sequences deposited in GenBank were incorrect.

Nevertheless, we agree that an alternate hypothesis is possible. The worm reported in our article could conceivably belong to a species that differs slightly from both *D. immitis* and *D. repens*, displaying morphologic similarities with *D. repens* but being more closely associated with *D. immitis* at the genomic level.
